# On embedding-based automatic mapping of clinical classification system: handling linguistic variations and granular inconsistencies

**DOI:** 10.1093/jamia/ocag004

**Published:** 2026-01-30

**Authors:** Santosh Purja Pun, Oliver Obst, Jim Basilakis, Jeewani Anupama Ginige

**Affiliations:** School of Computer, Data and Mathematical Sciences, Western Sydney University, Sydney, NSW 2196, Australia; School of Computer, Data and Mathematical Sciences, Western Sydney University, Sydney, NSW 2196, Australia; School of Computer, Data and Mathematical Sciences, Western Sydney University, Sydney, NSW 2196, Australia; School of Computer, Data and Mathematical Sciences, Western Sydney University, Sydney, NSW 2196, Australia

**Keywords:** clinical term embeddings, automatic mapping, clinical classification systems, large language models, prompting framework

## Abstract

**Objectives:**

Mapping clinical classification systems, such as the International Classification of Diseases (ICD), is essential yet challenging. While the manual mapping method remains labor-intensive and lacks scalability, existing *embedding-based* automatic mapping methods, particularly those leveraging transformer-based pretrained encoders, encounter 2 persistent challenges: (1) *linguistic variation* and (2) *varying granular details in clinical conditions*.

**Materials and Methods:**

We introduce an automatic mapping method that combines the representational power of pretrained encoders with the reasoning capability of large language models (LLMs). For each ICD code, we generate: (1) *hierarchy-augmented* (HA) and (2) *LLM-generated* (LG) descriptions to capture rich semantic nuances, addressing linguistic variation. Furthermore, we introduced a *prompting framework* (PR) that leverages LLM reasoning to handle granularity mismatches, including *source-to-parent* mappings.

**Results:**

Chapterwise mappings were performed between ICD versions (ICD-9-CM↔ICD-10-CM and ICD-10-AM↔ICD-11) using multiple LLMs. The proposed approach consistently outperformed the baseline across all ICD pairs and chapters. For example, combining HA descriptions with Qwen3-8B-generated descriptions yielded an average top-1 accuracy improvement of 6.5% (0.065) across the mapping cases. A small-scale pilot study further indicated that HA+LG remains effective in more challenging one-to-many mappings.

**Conclusions:**

Our findings demonstrate that integrating the representational power of pretrained encoders with LLM reasoning offers a robust, scalable strategy for automatic ICD mapping.

## Introduction

Disease classification systems such as the International Classification of Diseases (ICD) provide standardized codes for diseases and health conditions, facilitating accurate communication, reporting and analysis of health-care data globally. Clinical classification systems evolve over time into new versions, such as ICD-9, ICD-10, and the most recent ICD-11. In addition, countries often adapt these base classifications for local use, creating national extensions such as the United States’s ICD-10-CM (Clinical Modification), Canada’s ICD-10-CA (Canadian Adaptation), and Australia’s ICD-10-AM (Australian Modification). These continuous updates and country-specific modifications necessitate the development of mapping tables between classification systems to ensure consistency of previously coded data, enable longitudinal analysis, and support cross-country health data comparison.

There exist mapping tables for some ICD version pairs; however, they are constructed manually by domain experts. For example, the general equivalence mappings (GEMs),^1^ which provide crosswalk between ICD-9-CM and ICD-10-CM codes, were developed by the team of clinicians and coding experts. This manual mapping method is time-consuming. For instance, it took 3 years to develop the GEMs. Likewise, they are difficult to scale. The same mapping procedures must be followed to generate maps between any ICD version pairs for which the mapping tables do not exists (eg, ICD-9 to ICD-11 or ICD-10-AM to ICD-10-CA).

In recent years, the transformer-based language models have significantly advanced the field of natural language processing (NLP).^2^ Among them, pretrained encoder models such as BERT^3^ have emerged as powerful tools for generating discriminative dense representations of general text. These models have also demonstrated effectiveness in capturing meaningful representations of clinical terms.^4–7^ A natural extension of these methods to automate the mapping between ICD versions is to project the source and target ICD codes to a shared embedding space and identify potential source–target matching pairs using similarity metrics such as *cosine similarity.* We call this *embedding-based* automatic mapping method. While this approach yields promising results (see Appendix E), its effectiveness is fundamentally limited by 2 persistent challenges when mapping across ICD versions: (1) *linguistic variation* (eg, synonyms) and (2) *varying granular details in clinical conditions*.

To this end, we propose an automatic mapping approach that combines the representation capabilities of pretrained encoders with the reasoning abilities of large language models (LLMs). For each ICD code description, we (1) *generate a hierarchy-augmented* (HA) *description* and (2) *prompt a pretrained LLM to produce a concise clinical description* (LLM-generated [LG]). We encode these 2 descriptions separately, using a pretrained encoder model, and take their mean as the final embedding. To address variation in the level of detail across ICD code descriptions and ensure accurate mapping, we further leverage the reasoning capabilities of LLMs through a *prompting framework* (PR). We create a prompt in *multiple-choice* question format, asking the LLM to find the best match for a given source code description from a list of target code descriptions. It is important to note that the proposed method does not require any task-specific training or fine-tuning. It is model-agnostic and can be applied using any suitable pretrained models.

Empirically, we show the effectiveness of the proposed method by mapping different ICD versions, namely ICD-9-CM and ICD-10-CM, and ICD-10-AM and ICD-11. In this work, we opted for *chapterwise* mapping strategy, aligning equivalent chapters from the source and target ICD versions to facilitate the code-level mapping. Likewise, in this study, our primary focus is on *one-to-one* mapping (in the case of *one-to-many* relationships, we consider any partial match as complete). However, if the source concept is semantically broader in meaning than the any individual target codes, the union of multiple target concepts may approximate the source concept more closely (*one-to-many*). While we leave this for future work, we include a small-scale pilot study demonstrating that our method remains applicable in such cases.

Our main contributions are:

1. We identified and addressed 2 key challenges in automatic mapping of ICD versions, that is, *linguistic variation* and *varying granular details in clinical conditions*.
2. Our proposed method does not require any training (or fine-tuning) and does not rely on a specific family of pretrained models.
3. We empirically show the effectiveness of the proposed method by chapterwise mapping between ICD-9-CM↔ICD-10-CM and ICD-10-AM↔ICD-11 and demonstrate a significant gain in *top-1* accuracy compared to the chosen baseline.
4. Furthermore, we show that the method remains effective for more complex one-to-many mappings, as evidenced by a small-scale pilot study.

## Background and related works

### Background

**Figure 1.**
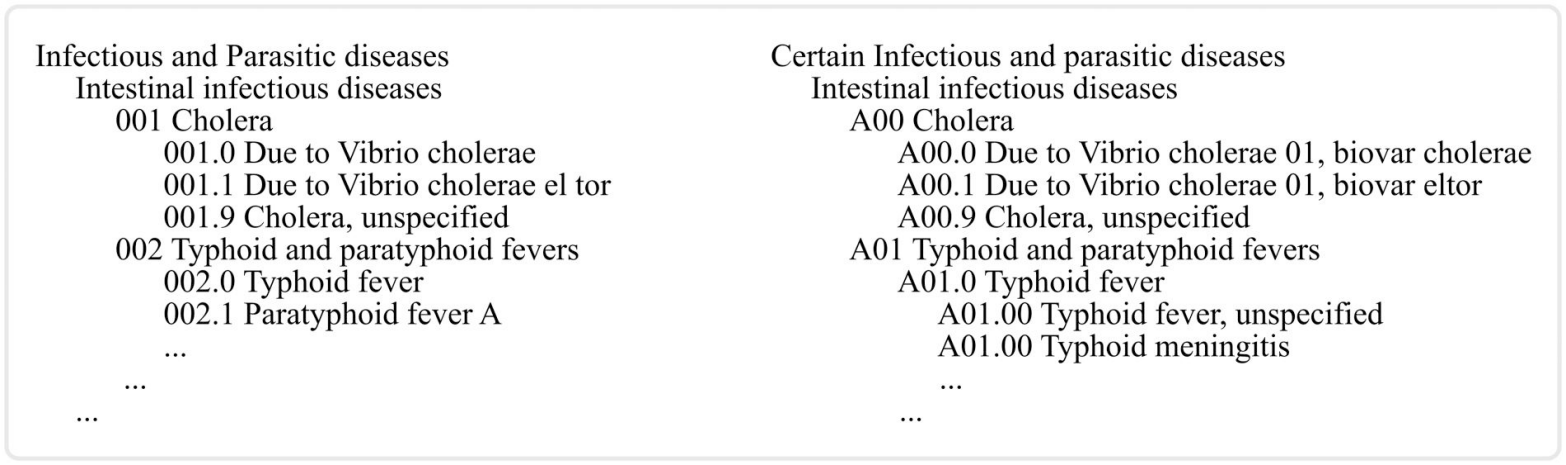
Comparison of code structures between ICD-9-CM (left) and ICD-10-CM (right) for the equivalent chapter on Infectious and parasitic diseases. Abbreviation: ICD, International Classification of Diseases.

The ICD is a hierarchical system that organizes clinical conditions into chapters, blocks, and groups based on various characteristics, such as affected body systems or causative agents. Figure 1 illustrates the code structure in ICD-9-CM and ICD-10-CM for an equivalent chapter (*Infectious and parasitic diseases*). Each condition is assigned a unique code with a brief description summarizing the clinical condition (*code description*). For example, ICD-9-CM assigns the code **001.0** for cholera infection due to *Vibrio cholerae*, whereas ICD-10-CM assigns **A00.0** for the same condition with a slight variation in the code description.

The different ICD versions are not directly comparable. For example, ICD-9 codes are mostly numeric, whereas ICD-10 codes are alphanumeric. International Classification of Diseases-11 codes are also alphanumeric, but they use completely different structures compared to ICD-10 codes. Likewise, the code descriptions are not uniform across the ICD versions. Different ICD versions may use different linguistic structures to describe equivalent clinical conditions.

While mapping between ICD versions is still predominantly a manual task performed by trained professionals with limited progress in automation, recent advancements, particularly in transformer-based encoders for representation learning, offer promising avenues. However, we identified 2 key challenges for implementing such *embedding-based* automatic mapping approaches: *linguistic variation* and *varying granular details in clinical conditions*. The following sections expand on the 2 challenges.

### Linguistic variation

The different ICD versions may use varying clinical terms (code descriptions) to describe the same condition. For instance, although ICD-9-CM code **073.0** (*Ornithosis with pneumonia*) and ICD-10-CM code **A70** (*Chlamydia psittaci infection*) are semantically equivalent, their clinical descriptions are linguistically distinct (see Appendix C for more examples). These terms are typically short and contain specialized vocabulary. As a result, due to limited contextual information, pretrained encoders often struggle to generate embeddings that accurately capture their semantic meanings.

### Varying granular detail in clinical conditions

Newer ICD versions are often more specialized than previous versions; therefore, they may define certain clinical conditions at a more granular level, incorporating distinctions based on specific causative agents or the presence or absence of complications. When mapping to a more specialized ICD version, parent codes in the target system sometimes share similar descriptions with codes in the source system. For example, while ICD-9-CM uses a single code (**002.0**) for *typhoid fever*, ICD-10-CM defines it in more granular levels, such as **A01.01** (*Typhoid meningitis*) and **A01.03** (*Typhoid pneumonia*). The parent for these codes in ICD-10-CM is **A01.0** (*Typhoid fever*). See Appendix D for more examples.

Conversely, *code consolidation* occurs when granular subclassifications that were once considered clinically relevant become obsolete. For example, ICD-9-CM distinguished between poliovirus types with separate codes: **045.20** (*unspecified type*), **045.21** (*type I*), **045.22** (*type II*), and **045.23** (*type III*) for acute nonparalytic poliomyelitis. These 4 codes were consolidated into a single ICD-10-CM code **A80.4** (*Acute nonparalytic poliomyelitis*), reflecting the diminished clinical utility of distinguishing between poliovirus strains in contemporary practice. Hence, when relying exclusively on code descriptions, the resulting embeddings for the source codes will exhibit a high degree of similarity with the parent codes; therefore, they are more likely to be mapped to the parent target code.

### Related works

Traditional *Lexical-based* mapping methods address *linguistic variation* among clinical terms by generating alternative terms, using existing knowledge sources such as SNOMED CT and WordNet.^8^^,^^9^ However, these approaches are fundamentally limited by their reliance on a simple *string-matching* technique to identify equivalent matches. More recent embedding-based methods have attempted to enhance clinical term representations by incorporating these alternative terms^4^^,^^10^; however, they remain constrained by the quality and coverage of the underlying knowledge sources. For instance, existing knowledge sources may fail to produce sufficient alternative terms for all ICD code descriptions.

Our work extends prior mapping approaches by leveraging LLMs to generate enriched term representations rather than depending solely on curated synonym lists from external ontologies. Specifically, we use LLMs to generate contextually rich descriptions and incorporate hierarchical information from code structures to address the linguistic variation.

Similarly, a closely related line of work is *ontology matching* (OM), which aligns entities across 2 distinct ontology systems to identify equivalent concepts. Ontology matching systems such as LogMap^11^ and BERTMap^12^ achieved strong performance by combining lexical similarity with structural propagation through hierarchical relationships. These approaches leverage the rich semantic information encoded in ontology structures, where parent–child and sibling relationships carry explicit semantic meaning (eg, subsumption, disjointness) that can guide the matching process. However, distinct challenges arise in ICD mapping. While ICD uses a hierarchical format, its structure is designed primarily for administrative grouping and statistical reporting rather than for strict semantic representation. Consequently, techniques that exploit ontological structure may not transfer effectively to ICD mapping, where hierarchical proximity does not consistently serve as a reliable proxy for semantic equivalence.

## Materials and methods

### Task definition

The ICD is a hierarchical coding system that organizes clinical conditions into chapters, blocks, and categories, assigning each condition a unique code. Formally, $C={\left\{(c_{i},\;d_{i},p_{i})\right\}}_{i}$, where $c_{i}$ is the *i*th code, $d_{i}$ is its corresponding textual description, and $p_{i}=\{p_{1},\;p_{2},\;\ldots ,\;p_{j}\}$ is the set of parent labels (eg, category and block labels) with $p_{1}$ being the immediate parent and $p_{j}$ being the root. Suppose $C_{\mathrm{src}}={\left\{(c_{i},d_{i},p_{i})\right\}}_{i}$ and $C_{\mathrm{tgt}}={\left\{(c_{j}^{\prime },d_{j}^{\prime },p_{j}^{\prime })\right\}}_{j}$ be the source and target ICD versions, respectively. Now, we define a mapping from source to target, $M_{\mathrm{src}\to \mathrm{tgt}}$, as follows:


(1) $$M_{\mathrm{src}\to \mathrm{tgt}}=\{(c_{i},\;c_{j}^{\prime })\;|\;c_{i}\in C_{\mathrm{src}},c_{j}^{\prime }\in C_{\mathrm{tgt}}\},$$

such that:

1. The description $d_{i}\;\mathrm{and}\;d_{j}^{\prime }\;\mathrm{describe}\;\mathrm{semantically}\;\mathrm{equivalent}\;\mathrm{clinical}\;\mathrm{concepts}\;\mathrm{for}\;\mathrm{each}\;(c_{i},c_{j}^{\prime })\;\in M_{\mathrm{src}\to \mathrm{tgt}}$.
2.
$\forall (c_{i},c_{j}^{\prime }),(c_{i},c_{k}^{\prime })\in M_{\mathrm{src}\to \mathrm{tgt}}:c_{j}^{\prime }=c_{k}^{\prime }$ (ie, each source code maps to at most 1 target code).

Due to differences in code granularity and scope across versions, mappings can take several forms, such as *one-to-one* (exact matches where codes represent identical concepts) and *one-to-many* mappings (when a broader source code maps to multiple specific target codes). While comprehensive ICD mapping systems must handle all these relationship types, this work primarily focuses on identifying *one-to-one* mappings, which constitute the majority of code correspondences and serve as the foundation for more complex mapping scenarios.

### Proposed method

**Figure 2.**
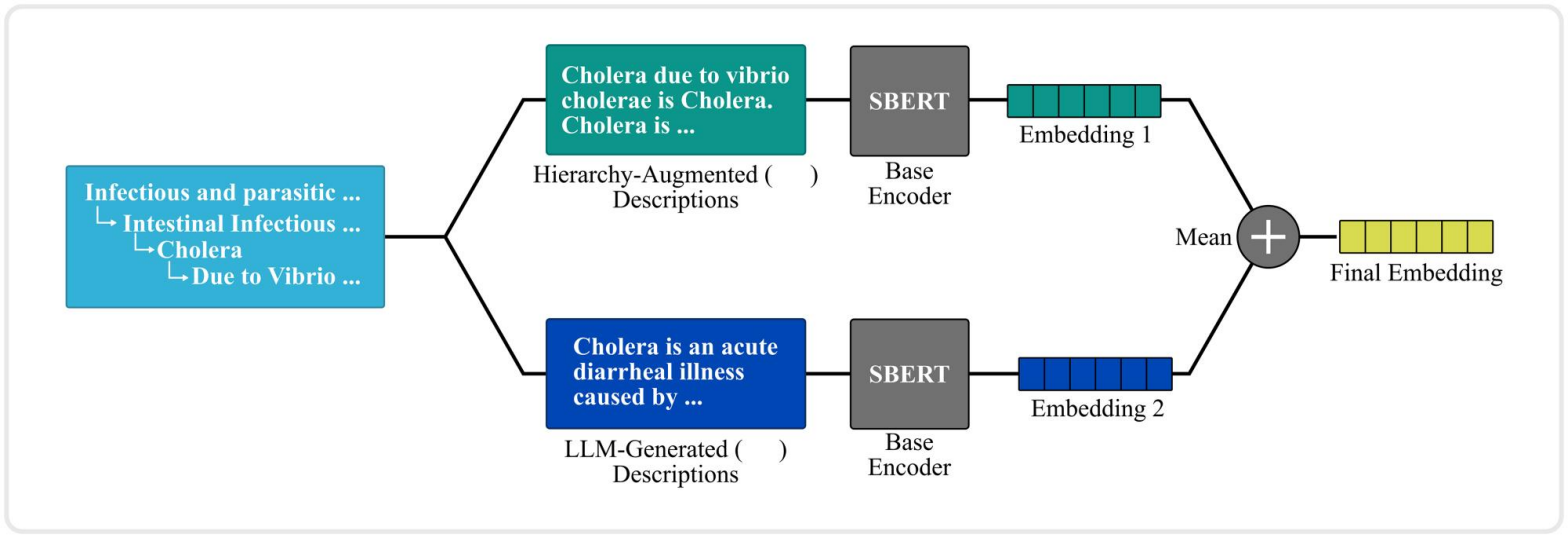
The overall process of generating dense representations for the ICD code descriptions. For each ICD codes, we generate: (1) a hierarchy-augmented (HA) description and (2) a concise description generated using a pretrained LLM (LG). Each description is encoded by an encoder model, and we take their mean as the final embedding. Abbreviations: ICD, International Classification of Diseases; LG, LLM-generated; LLM, large language model.

In this work, we aim to generate the mapping set by projecting both the source and target code descriptions into a shared embedding space, using a pretrained transformer-based encoders and use *cosine similarity* to identify semantically similar source–target code pairs in the embedding space. As discussed earlier, pretrained encoders fail to capture the semantic meanings of the ICD code descriptions due to the inherent *linguistic variation.* We aim to address this by generating: (1) an HA description and (2) a concise description generated using an LG. Figure 2 illustrates the overall process of generating the embeddings.

#### HA description

The HA variants utilize the structural context provided by a code’s position within the ICD hierarchy—specifically its parent or ancestor codes—to clarify and enrich the meaning of a code. To construct this, we concatenate the original code description with its hierarchical labels using the “is” relation to form a short, context-aware description as $d^{h}=$ “$[c_{i}]\;is[p_{i,\;1}]\ldots [p_{i,\;j-1}]\;is\;[p_{i,\;j}]”,\;$where $c_{i}\;$is the *i*th code and $p_{i,\;1},\ldots ,p_{i,\;j}\;$are its parent labels with $p_{i,\;1}$ being the immediate parent. For example, given the hierarchical structure for ICD-9-CM code **001.0** (ie, Intestinal infectious and parasitic diseases → Intestinal infectious disease → Cholera → Cholera due to *V. cholerae*), the HA description is: *Cholera due to V. cholerae is Cholera. Cholera is Intestinal infectious diseases. Intestinal infectious diseases is Infectious and parasitic diseases.*

This context-aware description is then encoded using a pretrained encoder model. In this work, we use the *Sentence-Transformer* (SBERT),^13^ specifically *all-mpnet-base-v2*, as the preferred encoding model:


(2) $$e^{h}=\mathrm{SBERT}(d^{h}).$$

#### LG description

Recent studies have demonstrated that fine-tuning models on synthetic data generated by LLMs can enhance performance across various downstream tasks, such as representation learning,^14^^,^^15^ fake news detection,^16^ and instance detection.^17^ Inspired by this, we generate a concise description for each code description using an LLM via a prompting method. This is particularly effective for reducing lexical variation, as LLMs tend to produce consistent outputs for similar prompts:


(3) $$e^{l}=\mathrm{SBERT}(d^{l}),\;\mathrm{and}\;d^{l}=\mathrm{LLM}(\mathrm{Prompt}(X)).$$

**Figure 3.**
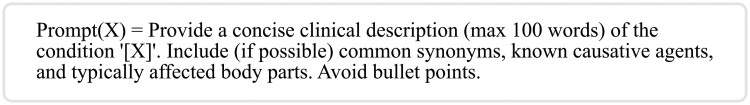
Prompt template to generate a concise description of an ICD code description. Here X is the placeholder for the code description. Abbreviation: ICD, International Classification of Diseases.

We construct prompts using a template (see Figure 3) and instruct a pretrained LLM to generate the concise description. The output is then encoded using SBERT. Finally, we compute the mean of the 2 embedding vectors (HA and LG) to obtain the final representation (ie, $e=\;1/2(e^{h}+e^{l})$). To evaluate the effectiveness of this approach, we conducted experiments using several open-source LLMs, *LLaMA-3.1-8B-Instruct,^18^ Qwen3-8B,^19^ Mistral-7B-Instruct-v0.3* (https://huggingface.co/mistralai/Mistral-7B-Instruct-v0.3), and *Microsoft-Phi-4-mini-Instruct.*^20^

#### Generating maps with PR

Given the source and the target code embeddings, the proposed method used *cosine similarity* score as the metric to find the potential maps:


(4) $$\underset{t_{i}^{*}=s_{i}\in S;t_{j}\in T}{\mathrm{argmax}}\cos\left(s_{i},\;t_{j}\right),\;j=1,\;2,\;...,\;n,$$

where $S=\;{\left\{s_{i}\right\}}_{i=1}^{m}$ and $T={\left\{t_{j}\right\}}_{j=1}^{n}$ are the set of source and target embeddings, respectively, and cos⁡(., .) is the cosine similarity between 2 vectors.

However, as discussed earlier some source codes get mapped to parent target codes, that is, *source-to-parent* mapping. Generally, these cases arise when mapping a less specialized version to a more specialized version. One approach to address these hierarchical inconsistencies is using a simple rule-based approach: *for each source-to-parent mapping cases select the immediate child code with highest cosine similarity scores.* Although this is simple and straightforward, this will always generate a map even if the source code is mapped to a wrong parent target code. It is crucial to identify these cases to enhance the reliability of the generated maps.

**Figure 4.**
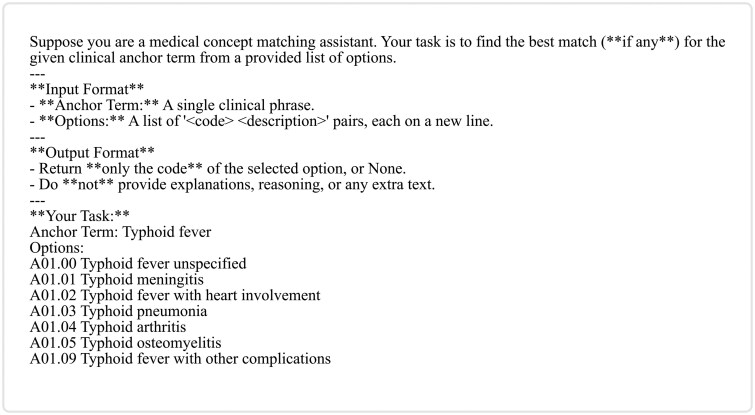
Example of a prompt used in PR for ICD-9-CM code 002.0 (Typhoid fever), which is mapped to parent ICD-10-CM code A01.0 (Typhoid fever), using an embedding-based method. The options are all the child codes of A01.0. Abbreviations: ICD, International Classification of Diseases; PR, prompting framework.

Recent studies have demonstrated the potential of LLMs in matching semantically similar entities.^21–23^ Building on this insight, we propose a prompting framework that leverages the reasoning ability of an LLM to generate the final mappings. For each case where a source code is mapped to a parent target code (eg, ICD-9-CM code **002.0** [*typhoid fever*] → ICD-10-CM code **A01.0 [***typhoid fever*]), we construct a *multiple-choice-style* prompt (see Figure 4). We use the source code description as the anchor term and all the child codes (of the mapped target parent code) as options. As shown in Figure 4, we explicitly instruct the model to either select only 1 valid option or reject all.

In this work, we use *Qwen3-8B* as the preferred LLM for PR because it allows a hard switch to enable the model’s thinking behavior. It is important to note that we only apply the rule-based prompts when the target code is not a leaf code; otherwise, we pick the code with the highest cosine similarity. Unlike data-driven approaches, where a model is trained explicitly on large volumes of labeled data, our method relies solely on the inherent ability of the LLM to process the prompts. This design choice underscores the importance of prompt quality,^24^ and we anticipate that developing more effective prompt strategies could further enhance PR performance, which we leave as a direction for future work.

### Experiment details

#### Dataset

We evaluated the proposed method by chapterwise mapping ICD-9-CM and ICD-10-CM and ICD-10-AM and ICD-11 across 3 chapters—*Infectious and parasitic Diseases* (**Inf**)*, Diseases of the Respiratory system* (**Resp**), *and Diseases of the Digestive system* (**Dig**). We used the ICD-9-CM (version 32) from the Centers for Medicare and Medicaid Services (CMS)^25^ and the ICD-10-CM (FY22 release) from the Centers for Disease Control and Prevention.^26^ Likewise, we used the ICD-10-AM (12th edition) provided by the Independent Health and Aged Care Pricing Authority (IHACPA).^27^ We accessed the ICD-11 codes via the WHO API (version 2.5) (https://icd.who.int/icdapi). The details on the number of mapping pairs across the different chapters and ICD version pairs are presented in Appendix B.

We used the GEMs provided by the CMS, as the ground-truth mappings between ICD-9-CM and ICD-10-CM. For ICD-10-AM and ICD-11, we used the sequential mapping^28^ approach. For ICD-10-AM to ICD-11, we first map ICD-10-AM to ICD-10 using the mapping tables provided by IHACPA,^29^ and then ICD-10 to ICD-11 using the conversion tables provided by the WHO (https://icd.who.int/browse/2025-01/mms/en). We followed the similar procedure to construct ICD-11 to ICD-10-AM. All datasets used in this study are publicly available.

#### Baseline method

For the baseline, we used only the ICD code descriptions to generate embeddings and applied a simple rule-based strategy to handle hierarchical inconsistencies. Specifically, for each source-to-parent mapping, the child code with the highest cosine similarity score was selected as the final mapping.

#### Models

We evaluated various transformer-based encoders (see Appendix E for details) with some specifically trained on the clinical data (*ClinicalBERT,^6^ BioClinicalBERT,*^7^ and *UMLSBert^5^*) and general text data (*SBERT*^13^). Compared to all other models, the *SBERT* (*all-mpnet-base-v2* [https://sbert.net/docs/sentence\_transformer/pretrained\_models.html]) performed significantly better, and hence we chose it as the preferred baseline encoder.

To validate the effectiveness of the proposed method, we experimented with various open-source LLMs, including *Llama-3.1-8B-Instruct*, *Mistral-7B-Instruct-v0.3*, *Phi-4-mini-instruct*, and *Qwen3-8B*, to generate the clinical descriptions. For the PR, we used *Qwen3-8B*. See Appendix A for the implementation details.

#### Evaluation metric

For evaluation metric, we report the *top-1* accuracy:


(5) $$\mathrm{Top}-1\;\mathrm{Accuracy}=\;\frac{C}{N-N_{nm}},$$

where C is the number of correct maps, N is the total number of source codes and $N_{nm}$ is the number of source codes that do not have any maps (some source codes do not have any maps in the ground-truth mapping files because they are either the immediate parent [eg, 3-digit codes] or are mapped to a different target chapter).

Given the inherent stochasticity of LLM-generated text, which introduces slight variations in output across multiple runs with the same prompt, we report the mean and SD of the *top-1* accuracy calculated from 5 independent runs for each prompt.

Likewise, for *one-to-many* mappings, we report:

1. Classwise mean Jaccard Similarity (JS) score:
  (6) $$\mathrm{JS}=\frac{1}{\left(N-N_{nm}\right)}.\sum_{i}\frac{P_{i}⋂G_{i}}{P_{i}⋃G_{i}},$$
    where $P_{i}\;\mathrm{and}\;G_{i}$ are the predicted and the ground-truth set of target codes for the *i*th source code.
2. Mapping Coverage (MC).
  (7) $$\mathrm{MC}=\frac{1}{\left(N-N_{nm}\right)}.|\{i\;|P_{i}\;\neq \;\emptyset \}|,$$
    where $P_{i}$ is the predicted set of target codes for the *i*th source code. Hence, mapping coverage is defined as *the proportion of source codes that have at-least 1 mapping to a target code*.

## Results

### Main results: chapterwise mapping

We present our main results in Table 1 for *chapterwise* mapping across different ICD versions. The baseline method exhibits consistent performance across all the mapping pairs and chapters with the top-1 accuracy ranging from 0.66 to 0.81. In contrast, our proposed method outperformed the baseline across all mapping pairs and chapters. And this is consistent across all evaluated LLMs to generate the clinical descriptions. For instance, incorporating HA with Qwen3-8B-LG resulted in an average top-1 accuracy improvement of 6.5% from 71.0% to 77.6%.

**Table 1.** Comparison of the proposed method against the baseline on *chapterwise* mapping of different ICD versions.

		ICD-9-CM to ICD-10-CM			ICD-10-CM to ICD-9-CM			ICD-10-AM to ICD-11			ICD-11 to ICD-10-AM		
		Dig	Inf	Resp	Dig	Inf	Resp	Dig	Inf	Resp	Dig	Inf	Resp
**Baseline**		0.81±0.0	0.72±0.0	0.77±0.0	0.70±0.0	0.70±0.0	0.66±0.0	0.71±0.0	0.68±0.0	0.72±0.0	0.66±0.0	0.73±0.0	0.66±0.0
**Proposed method**	**Qwen3-8B**	**0.88**±0.003	**0.80**±0.003	**0.82**±0.011	**0.81**±0.006	0.78±0.005	**0.74**±0.004	**0.74**±0.006	**0.72**±0.004	**0.80**±0.006	0.70±0.004	0.79±0.005	**0.74**±0.012
	**Llama-3.1-8B-Instruct**	0.87±0.004	**0.80**±0.005	0.80±0.009	0.80±0.013	0.78±0.006	0.73±0.007	0.72±0.01	**0.73**±0.004	0.78±0.01	**0.71**±0.006	**0.80**±0.012	0.73±0.016
	**Phi-4-mini-instruct**	0.87±0.003	0.79±0.002	0.80±0.0	0.80±0.002	0.77±0.0	**0.74**±0.002	0.73±0.003	0.72±0.004	0.78±0.003	0.69±0.004	0.79±0.001	**0.74**±0.003
	**Mistral-7B-Instruct-v0.3**	0.87±0.003	0.78±0.002	0.79±0.0	**0.81**±0.004	**0.79**±0.0	0.73±0.003	0.71±0.004	0.72±0.003	0.79±0.004	0.70±0.003	0.79±0.002	0.69±0.003

Dig, Inf, and Resp are, respectively, the Diseases of the Digestive System, the Intestinal Infectious Diseases, and the Diseases of the Respiratory System chapters. The numbers are the mean *top-1* accuracies and the SD after 5 runs. Bold values indicate best performance.

Abbreviation: ICD, International Classification of Diseases.

### Robustness and reliability

In Table 1, we also reported the SD to assess the consistency of the mapping performance across 5 runs. The proposed method, across all evaluated LLMs, demonstrated a high degree of consistency with very low SD, with the maximum being only *0.016*. These low SD values indicate that the performance gains of the proposed method are both robust and reliable.

### Handling linguistic variations

While code-only, HA, and LG descriptions achieved comparable performance across ICD chapters and mapping directions (see Appendix G), our qualitative analysis showed that LG descriptions effectively captured linguistic variation, enabling correct mappings even when baseline methods did not rank them within the top 100. For example, ICD-9-CM **073.0** (*Ornithosis with pneumonia*) was correctly mapped to ICD-10-CM **A70** (*Chlamydia psittaci infection*) using *Qwen3-8B-*generated descriptions (see Appendix F for additional examples).

### Evaluation of the PR

To evaluate the PR, we analyzed the *source-to-parent* mapping cases generated with HA+LG using *Qwen3-8B* in a single run. Table 2 shows the comparison between PR and simple-rule (SR)-based method. In 6 out of 12 cases, the PR method outperformed SR in generating correct maps (true positives) in *source-to-target* mapping cases with similar level of performance in 2 and slightly under performed in 3 cases. Notably, PR demonstrated an additional advantage by filtering out incorrect *source-to-parent* mappings (true negatives), thereby improving the overall mapping precision. A more detailed evaluation of PR, including statistics on incorrect selections (false positives) and incorrect rejections (false negatives) across 5 runs, is provided in Appendix K.

**Table 2.** Comparison between the *simple-rule* (SR) and the *prompting framework* (PR) in handling the *source-to-parent* mapping cases.

	Chapters	True positive		True negative	
		SR (%)	PR (%)	SR (%)	PR (%)
**ICD-9-CM to ICD-10-CM**	Dig (64)	57.81	**62.5**	—	**12.5**
	Inf (74)	63.51	**74.32**	—	**5.4**
	Resp (19)	52.63	**52.63**	—	0.0
**ICD-10-CM to ICD-9-CM**	Dig (81)	70.37	77.78	—	**6.17**
	Inf (0)	0.0	0.0	—	0.0
	Resp (37)	54.05	54.05	—	**13.51**
**ICD-10-AM to ICD-11**	Dig (47)	**51.06**	44.68	—	**6.38**
	Inf (73)	**32.88**	31.5	—	**5.48**
	Resp (23)	26.09	**47.82**	—	**4.35**
**ICD-11 to ICD-10-AM**	Dig (70)	**44.28**	30	—	**14.29**
	Inf (68)	39.70	**63.23**	—	**5.88**
	Resp (16)	25.0	**31.25**	—	**18.75**

Results show proportion of correct selections (true positive) and correct rejections (true negative) cases for the HA+LG method with *Qwen3-8B* in a single run. The total number of *source-to-parent* mapping cases are shown in parentheses for each chapter. Bold values indicate best performance; underlined values indicate equivalent performance.

### One-to-many mappings

To highlight the effectiveness of the proposed method (ie, HA+LG) in handling the more challenging *one-to-many* mappings, we conducted a small-scale pilot study using 2 simple strategies (for LG, we used the clinical descriptions generated using Qwen3-8B): a *threshold-based* method, which includes all target codes with cosine similarity scores above a predefined threshold value (*λ*), and a *top-K* selection method, which selects the *K* target codes with the highest cosine similarity scores for each source code. As shown in Tables 3 and 4, the proposed method outperformed the baseline, across both strategies, highlighting the significance of the method in handling these complex mapping scenarios.

**Table 3.** Comparison between the baseline and the proposed method (HA+LG) on *one-to-many* mappings, with *λ *= 0.90.

		ICD-9-CM to ICD-10-CM		ICD-10-CM to ICD-9-CM		ICD-10-AM to ICD-11		ICD-11 to ICD-10-AM	
		JS	MC	JS	MC	JS	MC	JS	MC
**Dig**	**Baseline**	0.5827	0.75	0.5715	0.62	**0.6872**	0.65	0.7423	0.44
	**HA+LG**	**0.6567**	**0.82**	**0.6525**	**0.64**	0.6794	**0.69**	**0.7579**	**0.46**
**Inf**	**Baseline**	0.76	0.5	0.8421	0.43	0.6912	0.6	0.7503	0.58
	**HA+LG**	**0.7722**	**0.55**	0.8411	**0.47**	0.7241	**0.64**	**0.761**	**0.63**
**Resp**	**Baseline**	0.7006	0.7	0.6792	**0.59**	0.7512	0.69	**0.8333**	0.58
	**HA+LG**	**0.7725**	**0.7**	**0.7448**	0.54	0.7312	**0.72**	0.8058	**0.59**

Results are reported as classwise mean *Jaccard Similarity* (JS) and *Mapping Coverage* (MC). Bold values indicate best performance.

**Table 4.** Comparison between the baseline and the proposed method (HA+LG) on one-to-many mappings using top-K selection strategy.

		ICD-9-CM to ICD-10-CM			ICD-10-CM to ICD-9-CM			ICD-10-AM to ICD-11			ICD-11 to ICD-10-AM		
		Dig	Inf	Resp	Dig	Inf	Resp	Dig	Inf	Resp	Dig	Inf	Resp
**K = 3**	**Baseline**	0.2768	0.2755	0.2768	0.2648	0.2832	0.2648	0.2876	0.2683	0.2876	0.2443	0.2733	0.2443
	**HA+LG**	**0.2976**	**0.3024**	**0.2976**	**0.2772**	**0.3076**	**0.2772**	**0.3072**	**0.2871**	**0.3072**	**0.2621**	**0.2822**	**0.2621**
**K = 5**	**Baseline**	0.1779	0.1777	0.1779	**0.1895**	0.1781	**0.1895**	0.1874	0.1785	0.1874	0.1573	0.1726	0.1573
	**HA+LG**	**0.1929**	**0.1944**	**0.1929**	0.1851	**0.1977**	0.1851	**0.1926**	**0.1889**	**0.1926**	**0.1689**	**0.1855**	**0.1689**
**K = 7**	**Baseline**	0.1386	0.1331	0.1386	**0.1396**	0.131	**0.1396**	0.1372	0.132	0.1372	0.1158	0.1296	0.1158
	**HA+LG**	**0.1435**	**0.1457**	**0.1435**	0.138	**0.1445**	0.138	**0.1398**	**0.1396**	**0.1398**	**0.1248**	**0.1379**	**0.1248**

Results are reported as classwise mean *Jaccard Similarity* (JS). Mapping Coverage (MC) is not reported, as top-K selection always produces a mapping for all source codes. Bold values indicate best performance.

## Discussion

While the prompting framework demonstrated the ability to select correct mappings and filter out some incorrect source-to-parent instances, certain errors remained in the form of incorrect selections (false positives) and false rejections (false negatives). Detail statistics of these cases are presented in Appendix K. To further assess the effectiveness of the prompting framework, we conducted an analysis of these error cases and identified several recurring patterns.

Our analysis of the false positive cases highlighted 2 key trends. First, we observed inconsistencies in the ground-truth mappings—specifically, instances where source codes were explicitly mapped to the parent codes. For example, in ICD-9-CM→ICD-10-CM mappings for the Disease of the Digestive System chapter, 11 such cases were identified, out of which the PR selected one of the child codes in 8 cases (Appendix I). In all these cases, the model opted for the most general option as a match. One potential strategy to address this is by adjusting the ground-truth mappings, such as by distributing parent mappings across all corresponding child codes. However, we argue that expert validation of these cases is essential to provide a more accurate reflection of the true performance gains.

Second, several false positives resulted from incorrect source-to-parent mappings, where the model selected an option rather than rejecting all the options. These cases typically occurred when mapping from a newer to an older ICD version. For example, there were 8 such instances out of 11 when mapping from ICD-10-CM to ICD-9-CM within the Diseases of the Digestive System chapter (Appendix J). In these instances, the model opted for the most general option that partially matched the anchor term. For example, for ICD-10-CM code K38.2 (Diverticulum of appendix), the model incorrectly selected ICD-9-CM 562.1 (Diverticula of colon), overlooking the anatomical distinction between the appendix and the colon.

Conversely, false negatives often occurred when the model rejected all options despite the presence of a valid, albeit more general, match. When the anchor term was broad (eg, *typhoid fever*), the model correctly selected the most general option (eg, *typhoid fever, unspecified*). However, when the anchor was specific (eg, *gonococcal endophthalmitis*), the model often rejected all the options, even when a broader but clinically relevant match (eg, *other gonococcal eye infection*) was available. This dual behavior—selecting general options in some cases while rejecting all candidates in others—suggests that the model does not apply a uniform decision strategy across mapping scenarios. Instead, its reasoning appears to be context-sensitive, influenced by the specificity of the anchor term and the semantic granularity of the available options.

### Future directions

Some codes are residual categories, serving as general options when a condition cannot be classified more specifically. For instance, ICD-10-CM code **A00.9** (*Cholera*, *unspecified*) is used only if a condition cannot be classified as either **A00.0** (*Cholera due to V. cholerae 01, biovar cholerae*) or **A00.1** (*Cholera due to V. cholerae 01, biovar eltor*). Simply generating a generic description for these codes may overlook their intended role in the hierarchy. Future works could explore methods to explicitly include these facts in the description generation process for such residual to improve the quality of the generated embedding, thereby enhancing the overall mapping accuracy.

Prior studies have shown that well-crafted prompts can substantially enhance the reasoning capabilities of LLMs without additional fine-tuning.^30^^,^^31^ Motivated by this, we conducted a small exploratory case study on selected instances of false negatives and false positives. By slightly modifying the original prompt and adding 3 simple rules (Appendix H), we observed improved reasoning consistency—particularly in correcting false negative cases. However, performance on false positives remained inconsistent. These findings highlight the potential of effective prompt design. Future work could explore more systematic and adaptive prompt design strategies to enhance both the accuracy and consistency of mappings across diverse clinical concepts.

This study relies solely on existing mapping files for evaluation. However, given the inconsistencies in these manually curated mappings and their varied use cases, expert evaluation remains essential. For example, our ICD-10-AM↔ICD-11 evaluation used ICD-10 as an intermediate reference version (ICD-10-AM→ICD-10→ICD-11), which may introduce transitivity issues. For instance, given that ICD-10-AM is more granular than ICD-10, it would result in parent codes being mapped to target ICD-11 codes or vice versa. While our results demonstrate the proposed method’s capabilities under these challenging conditions, direct expert validation would provide a more definitive assessment of mapping correctness and clinical utility. Beyond evaluation, expert feedback offers opportunities for model improvement through techniques such as, fine-tuning the model through *Reinforcement Learning from Human Feedback*^32–34^ or its alternatives like *Direct Preference Optimization*,^35^ in which expert input iteratively refines the model’s reasoning and mapping behavior to align with domain-specific expectations.

The proposed method could be deployed as a decision-support tool to help clinical coding experts create and validate cross-version mappings. Rather than fully automated mapping, the system could generate a ranked list of top-K candidate target codes for each source code, which domain experts would then review to select the most appropriate mapping(s) or reject all candidates if none are suitable. This human-in-the-loop approach would combine the efficiency of automated candidate generation with the domain expertise and clinical judgment necessary for high-quality mappings, particularly for complex or ambiguous cases. Such a system could significantly reduce the manual effort required to create comprehensive mapping files while maintaining the accuracy and reliability needed for clinical and administrative applications.

Finally, we demonstrated the effectiveness of the proposed method (HA+LG) in handling one-to-many mapping cases. However, such cases remain inherently more challenging. Future work could extend the PR to address these scenarios. One potential direction is to adopt a *filtering-and-matching* pipeline, commonly used in *ontology matching* tasks,^12^^,^^36^^,^^37^ where embeddings generated using HA+LG could first be used to identify a set of candidate target codes. Afterward an LLM could be prompted to select all potential matches from among these candidates. We leave a detailed investigation of this approach as part of our future work.

## Conclusion

In summary, we identified 2 key limitations of embedding-based ICD mapping methods—linguistic variation and differing clinical granularity—and proposed a hybrid approach that combines transformer-based encoders with LLM reasoning. By incorporating HA and LG descriptions along with a PR framework, our method captures fine-grained semantics and manages granularity differences. Empirical evaluation on chapterwise mappings between ICD-9-CM↔ICD-10-CM and ICD-10-AM↔ICD-11, across 3 different chapters, shows a consistent improvement over the chosen baseline method.

## Supplementary Material

ocag004_Supplementary_Data

## Data Availability

All datasets used in this study, including processed ICD code descriptions, ground-truth mappings, LLM-generated descriptions, and the raw embeddings, will be made publicly available via Dryad. The source code used to implement the proposed mapping framework is provided in the Supplementary Material. Detailed documentation will be included to support reproducibility and reuse.
