# Supplementary material for: On embedding-based automatic mapping of clinical classification system: handling linguistic variations and granular inconsistencies
Source: J Am Med Inform Assoc. 2026 Jan 30;33(4):792–801. doi: 10.1093/jamia/ocag004 (PMC13089492; doi:10.1093/jamia/ocag004)
Supplement: ocag004_Supplementary_Data [file ocag004_supplementary_data.zip › appendix.docx]

**APPENDIX**

**A. Implementation Details**

**B. Dataset Details**

**C. ICD-9-CM and ICD-10-CM equivalent codes but with different linguistic structures**

**D. Examples of varying granular details in clinical conditions across different ICD versions**

**E. Evaluation of various BERT models for mapping ICD versions**

**F. Evaluation of LLM generated descriptions (LG) for handling linguistic variation**

**G. Comparison of the ICD version mapping performance using different description types.**

**H. Effectiveness of Explicitly Including Rules in the Prompt – A Case Study**

**I. Examples of False Positive Cases Due to Explicit Source-to-Parent Mappings in the Ground Truth for ICD-9-CM to ICD-10-CM within the Disease of the Digestive System Chapter**

**J. Examples of the False Positive Cases Due to Incorrect Selection Instead of Rejecting All Options for ICD-10-CM to ICD-9-CM within the Disease of the Digestive System Chapter**

**K. Detailed Evaluation of the Prompting Framework.**

1. **Implementation details**

We ran all our experiments on a single Nvidia A30 GPU with *cuda-12.6*, and our source code is implemented in Python 3.11. We used Huggingface's *transformer-v4.51.3* to load the LLMs. We used *sentence-transformers-v4.1.0* to load the SBERT.

For the clinical description generation (LG) task, we used the default values for all the hyperparameters, for example, *temperature*=1.0 and *do_sample*=False, and set the *max_new_tokens*=512}. And for the reasoning task, we set *max_new_tokens*=32768 and *enable_thinking*=True} when applying the chat template.

1. **Dataset details**

We employed a chapter-wise mapping strategy, concentrating on the Infectious and parasitic Diseases (**Inf**), Diseases of the Respiratory system (**Resp**) and Diseases of the Digestive system (**Dig**) chapters. We used this approach to limit the search space for the potential maps. Also, we include all the three- and four-digit codes. Hence, as shown in Table 1, several codes have no maps because they are either the immediate parents or a broader category in the hierarchy. Additionally, there are also cases where source codes are either partially or full mapped to different target chapters. Table 2 shows the percentage of these cases out of the total valid mappings as per the ground truth. For partial cases, we just exclude the target codes in different chapters and use the in-chapter codes as the valid mappings, whereas in the case of full mappings to different chapters, we exclude those cases.

Table 1. Total number of codes **(N)**, and the number of cases where the source code has no maps in ground truth mapping files (**N_nm_**)

| **Chapters** | **ICD-9-CM to ICD-10-CM** | | **ICD-10-CM to ICD-9-CM** | | **ICD-10-AM to ICD-11** | | **ICD-11 to ICD-10-AM** | |
| --- | --- | --- | --- | --- | --- | --- | --- | --- |
|  | ***N*** | ***N_nm_*** | ***N*** | ***N_nm_*** | ***N*** | ***N_nm_*** | ***N*** | ***N_nm_*** |
| **Dig** | 757 | 161 | 795 | 190 | 617 | 164 | 969 | 421 |
| **Inf** | 889 | 0 | 1158 | 110 | 921 | 156 | 1004 | 347 |
| **Resp** | 320 | 65 | 369 | 61 | 281 | 58 | 342 | 126 |

Table 2. Percentage of cases where the source codes are mapped either partially or fully to different target chapters, out of total valid cases as per the ground truth mappings.

|  | **ICD-9-CM to ICD-10-CM** | | **ICD-10-CM to ICD-9-CM** | | **ICD-10-AM to ICD-11** | | **ICD-11 to ICD-10-AM** | |
| --- | --- | --- | --- | --- | --- | --- | --- | --- |
|  | **Partial (%)** | **Full (%)** | **Partial (%)** | **Full (%)** | **Partial (%)** | **Full (%)** | **Partial (%)** | **Full (%)** |
| **Dig** | 3.5 | 18.95 | 0.66 | 3.8 | 4.41 | 27.81 | 1.28 | 2.92 |
| **Inf** | 2.07 | 0 | 5.24 | 0.67 | 0.91 | 6.67 | 0.76 | 7.76 |
| **Resp** | 1.25 | 8.75 | 2.92 | 3.57 | 0 | 7.17 | 0 | 4.63 |

1. **ICD-9-CM and ICD-10-CM equivalent codes but with different linguistic structures**

Table 3. ICD-9-CM and ICD-10-CM equivalent codes but with different linguistic structures.

| **ICD-9-CM** | **ICD-10-CM** |
| --- | --- |
| Madura foot [**039.4**] | Mycetoma unspecified [**B47.9**] |
| Ornithosis with pneumonia [**073.0**] | Chlamydia psittaci infection [**A70**] |
| Herpangina [**074.0**] | Enteroviral vesicular pharyngitis [**B08.5**] |
| Condyloma acuminatum [**078.11**] | Anogenital (venereal) warts [**A63.0**] |
| Toxocariasis [**128.0**] | Visceral larva migrans [**B83.0**] |
| Pneumoconiosis due to other inorganic dust [**503**] | Stannosis [**J63.5**] |

1. **Examples of varying granular details in clinical conditions across different ICD versions**

Table 4. Examples of cases where source and target code descriptions are similar, but the target system defines the clinical condition in more granular sub-codes with * indicating the actual mapped target code.

| **Source** | **Target** |
| --- | --- |
| **006.8** Amebic infection of other sites [ICD-9-CM] | **A06.8** Amebic infection of other sites [ICD-10-CM] |
|  | **A06.81** Amebic cystitis |
|  | **A06.82** Other amebic genitourinary infections |
|  | **A06.89** Other amebic infections^*^ |
| **112.89** Other candidiasis of other specified sites [ICD-9-CM] | **B37.8** Candidiasis of other sites [ICD-10-CM] |
|  | **B37.81** Candidal esophagitis |
|  | **B37.82** Candidal enteritis |
|  | **B37.83** Candidal cheilitis |
|  | **B37.84** Candidal otitis externa |
|  | **B37.89** Other sites of candidiasis^*^ |
| **A48.3** Toxic shock syndrome [ICD-10-AM] | **1C45** Toxic shock syndrome [ICD-11] |
|  | **1C45.0** Streptococcal toxic shock syndrome |
|  | **1C45.1** Staphylococcal toxic shock syndrome |
|  | **1C45.Y** Toxic shock syndrome due to other specified infectious agent |
|  | **1C45.Z** Toxic shock syndrome without specified infectious agent^*^ |
| **B56.0** Gambiense trypanosomiasis [ICD-10-AM] | **1F51.0** Gambiense trypanosomiasis, [ICD-11] |
|  | **1F51.00** Meningitis in gambiense trypanosomiasis |
|  | **1F51.0Y** Other specified gambiense trypanosomiasis |
|  | **1F51.0Z** Gambiense trypanosomiasis, unspecified^*^ |

1. **Evaluation of various BERT models for mapping ICD versions**

We evaluated multiple pre-trained BERT models to generate dense vector representations of ICD code descriptions. ClinicalBERT, BioClinicalBERT, and UmlsBERT are trained specifically on clinical texts. *Sentence-Transformer* (SBERT) provides a set of models trained on general text to generate sentence-level embeddings. In this work, we used *all-mpnet-base-v2* as the SBERT encoder. *Table 5* reports the mapping accuracy achieved by each model. Interestingly, SBERT consistently outperformed all other models.

Table 5. Evaluation of various pre-trained BERT models for mapping between different ICD versions. These models are used to generate the embeddings for the ICD code descriptions, and the potential maps are identified using cosine-similarity.

|  | **ICD-9-CM to ICD-10-CM** | | | **ICD-10-CM to ICD-9-CM** | | | **ICD-10-AM to ICD-11** | | | **ICD-11 to ICD-10-AM** | | |
| --- | --- | --- | --- | --- | --- | --- | --- | --- | --- | --- | --- | --- |
|  | **Dig** | **Inf** | **Resp** | **Dig** | **Inf** | **Resp** | **Dig** | **Inf** | **Resp** | **Dig** | **Inf** | **Resp** |
| **BioClinicalBERT** | 0.67 | 0.57 | 0.63 | 0.51 | 0.53 | 0.55 | 0.58 | 0.53 | 0.63 | 0.43 | 0.56 | 0.57 |
| **ClinicalBERT** | 0.73 | 0.60 | 0.68 | 0.53 | 0.56 | 0.58 | 0.63 | 0.57 | 0.67 | 0.46 | 0.57 | 0.60 |
| **UmlsBERT** | 0.75 | 0.58 | 0.67 | 0.53 | 0.56 | 0.55 | 0.62 | 0.56 | 0.65 | 0.47 | 0.57 | 0.54 |
| **SBERT** | **0.80** | **0.69** | **0.75** | **0.62** | **0.70** | **0.59** | **0.66** | **0.66** | **0.71** | **0.60** | **0.67** | **0.61** |

1. **Evaluation of LLM generated descriptions (LG) for handling linguistic variation**

We analysed the generated maps using LLM-generated descriptions and terms-only, to evaluate the effectiveness of the LLM-generated descriptions at capturing the linguistic variation in the code description across different ICD versions. we identified cases where the ground-truth target codes did not appear among the top-100 predicted mappings when using only the code descriptions but were correctly retrieved when using the summaries generated by *Qwen3-8B*. *Table 6* presents some examples of such cases. This suggests that LLM-generated texts do provide meaningful context to generate better embeddings.

Table 6. Examples of cases where ground-truth target codes were not in the Top-100 predictions using the ICD code descriptions but correctly mapped using summaries generated by Qwen3-8B.

| **ICD-9-CM** to **ICD-10-CM** | |
| --- | --- |
| **Source** | **Target** |
| Madura Foot [039.4] | Mycetoma unspecified [B47.9] |
| Geniculate Herpes Zoster [053.11] | Postherpetic geniculate ganglionitis [B02.21] |
| Ornithosis with pneumonia [073.0] | Chlamydia psittaci infection [A70] |
| Condyloma acuminatum [078.11] | Anogenital (venereal) warts[A63.0] |
| Hand, foot, and mouth disease [074.3] | Enteroviral vesicular stomatitis with exanthem [B08.4] |
| Blood in stool [578.1] | Melena[K92.1] |
| **ICD-10-CM** to **ICD-9-CM** | |
| Enteroviral vesicular pharyngitis [B08.5] | Herpangina [074.0] |
| Tinea cruris [B35.6] | Dermatophytosis of groin and perianal area [110.3] |
| Naegleriasis [B60.2] | Other specific infections by free-living amebae [136.29] |
| Cercarial dermatitis [B65.3] | Cutaneous schistosomiasis [120.3] |
| Visceral larva migrans [B83.0] | Toxocariasis [128.0] |
| Stannosis [J63.5] | Pneumoconiosis due to other inorganic dust [503] |
| **ICD-10-AM** to **ICD-11** | |
| Necrotising ulcerative stomatitis [A69.0] | Necrotising ulcerative gingivitis, unspecified [1C1H.Z] |
| Toxoplasma oculopathy [B58.0] | Eye disease due to toxoplasma gondii [1F57.3] |
| Exanthema subitum (sixth disease) [B08.2] | Roseola infantum [1F01] |
| Tinea unguium [B35.1] | Dermatophytosis of nail [1F28.1] |
| Mottled teeth [K00.3] | Fluoride related opacities or lesions [DA07.0] |
| Hypercementosis [K03.4] | Cementum dysplasia [DA07.5] |
| **ICD-11** to **ICD-10-AM** | |
| Postdiphtheritic paralysis of uvula [1C17.00] | Pharyngeal diphtheria [A36.0] |
| Disseminated lyme borreliosis, unspecified [1C1G.1Z] | Lyme disease [A69.2] |
| Candidosis of lips or oral mucous membranes [1F23.0] | Candidal stomatitis [B37.0] |
| Dermatophytosis of foot [1F28.2] | Tinea Pedis [B35.3] |
| Talaromycosis [1F2K] | Penicilliosis [B48.4] |
| Burning mouth syndrome [DA0F.0] | Glossodynia [K14.6] |

1. **Comparison of the ICD version mapping performance using different description types.**

We evaluated the quality of the generated maps using the embeddings obtained from different methods—using only the clinical descriptions, hierarchy-augmented (**HA**) descriptions and the LLM-generated (**LG**) descriptions. As shown in *Table 7* , using these different descriptions yielded comparable performance across different chapters and ICD version pairs.

Table 7. Comparison of the ICD version mapping performance using different description types. **Terms-Only** uses only the ICD code descriptions (Baseline)}. **HA** and **LG** use hierarchical-augmented description and LLM-generated description, respectively. Results are presented for specific chapters, across different ICD version mapping pairs. The numbers are Mean Top-1 Accuracy ± Standard Deviation over five runs.

|  | | **Dig** | **Inf** | **Resp** |
| --- | --- | --- | --- | --- |
| **Terms-only** | | 0.67 (±0.0) | **0.68 (**±0.0) | 0.67 (±0.0) |
| **HA** | | 0.66 (±0.0) | **0.68 (**±0.0) | **0.68 (**±0.0) |
| **LG** | **Qwen3-8B** | **0.68 (**±0.058) | **0.68 (**±0.02) | **0.68 (**±0.038) |
|  | **Llama-3.1-8B-Instruct** | 0.64 (±0.041) | 0.66 (±0.023) | 0.63 (±0.04) |
|  | **Phi-4-mini-instruct** | 0.65 (±0.0) | 0.66 (±0.0) | 0.65 (±0.0) |
|  | **Mistral-7B-Instruct -v0.3** | 0.64 (±0.0) | 0.66 (±0.0) | 0.66 (±0.0) |

1. **Effectiveness of Explicitly Including Rules in the Prompt – A Case Study**

The goal of this case study is to highlight the importance of prompt design to enhance the reasoning capabilities of the LLM. For this, we took the false negative cases (i.e., cases in which all options were incorrectly rejected) and the false positive cases, where the model should have rejected all the options across multiple different runs—at least 3 out of 5 instances. We slightly modified the prompt and explicitly include three rules to guide the LLM in choosing the best matching option (see for an example). Specifically, we added the following three rules:

1. Reject any option that doesn't match the *specific condition* of the Anchor Term.
2. Select the option that directly aligns with the Anchor Term.
3. When both “other” and “unspecified” options are available:
4. Choose “other” if the Anchor Term specifies a particular subtype or manifestation not explicitly listed
5. Choose “unspecified” if the Anchor Term is vague or lacks detail.

To evaluate the effectiveness of the modified rule-based prompts, we ran each prompt five times and explicitly instruct the model to briefly include the rule it used to select a particular answer. *Table 8* reports the results for each of the test cases. The results show that the LLM consistently applied relevant rule to select the correct option. However, on the false positive cases the results were inconsistent. For example, for *Gingival and edentulous alveolar ridge lesions associated with trauma,* the model correctly rejected all the options on 4 (out of 5) times. While for *Other cholelithiasis with obstruction* and *Gastrostomy hemorrhage*, the model consistently opted for a general option rather than rejecting all the options in both cases.

**Role and Goal: ** You are a medical concept matching assistant. Your task is to find the best match for a given clinical anchor term from a provided list of options. Use the following **Rules** for guidance.

---

**Rules**

1. Reject any option that doesn't match the **specific condition** of the Anchor Term.

2. Select the option that directly aligns with the Anchor Term.

3. When both "other" and "unspecified" options are available:

a. Choose "other" if the Anchor Term specifies a particular subtype or manifestation not explicitly listed.

b. Choose "unspecified" if the Anchor Term is vague or lacks detail.

---

**Input Format**

- **Anchor Term: ** A single clinical phrase.

- **Options: ** A list of `<code> <description>` pairs, each on a new line.

---

**Output Format**

- Return **only the code** of the selected option, or `None`, with a brief rationale stating which rule(s) triggered the selection.

**Your Task: **

Anchor Term: Gonococcal endophthalmia

Options:

A54.30 Gonococcal infection of eye unspecified

A54.31 Gonococcal conjunctivitis

A54.32 Gonococcal iridocyclitis

A54.33 Gonococcal keratitis

A54.39 Other gonococcal eye infection

Figure 1. Example of rule-based prompt template for the anchor term Gonococcal endophthalmia.

Table 8. Evaluation of the effectiveness of explicitly including rules in the prompts, using selected false negative and false positive. For each sample, the prompt was executed five times, and the number in the parenthesis, i.e., (), denotes the number of times the model selected the corresponding option.

| **Source** | **Options** | **Ground Truth** | **Predicted** | **Rationale** |
| --- | --- | --- | --- | --- |
| Syphilitic parkinsonism | A52.10 Symptomatic neurosyphilis unspecified  A52.11 Tabes dorsalis  A52.12 Other cerebrospinal syphilis  A52.13 Late syphilitic meningitis  A52.14 Late syphilitic encephalitis  A52.15 Late syphilitic neuropathy  A52.16 Charcôt's arthropathy (tabetic)  A52.17 General paresis  A52.19 Other symptomatic neurosyphilis | A52.19 | A52.19 (5) | The anchor term "syphilitic parkinsonism" refers to a specific subtype of neurosyphilis not explicitly listed in the options. According to Rule 3a, when the anchor term specifies a particular subtype not explicitly listed, "other" should be selected. A5219 ("Other symptomatic neurosyphilis") is the most appropriate code as it covers unspecified or less common forms of neurosyphilis, including parkinsonism. |
| Gonococcal endophthalmia | A54.30 Gonococcal infection of eye unspecified  A54.31 Gonococcal conjunctivitis  A54.32 Gonococcal iridocyclitis  A54.33 Gonococcal keratitis  A54.39 Other gonococcal eye infection | A54.39 | A54.39 (5) | The anchor term "gonococcal endophthalmia" refers to a specific subtype of gonococcal eye infection not explicitly listed in the options. According to Rule 3a, when the anchor term specifies a particular subtype not included in the options, "other" (A5439) is the correct choice. |
| Chronic meningococcemia | 1C1C.20 acute meningococcaemia  1C1C.2Y other specified meningococcaemia  1C1C.2Z meningococcaemia, unspecified | 1C1C.2Y | 1C1C.2Y (5) | The anchor term "Chronic meningococcemia" specifies a particular subtype (chronic) not explicitly listed in the options. According to Rule 3a, "other specified" (code 1C1C2Y) is the correct choice when the term indicates a specific subtype not included in the available options. |
| Cytomegaloviral pneumonitis | 1D82.0 cytomegaloviral hepatitis  1D82.1 cytomegaloviral pancreatitis  1D82.Y other specified cytomegaloviral disease  1D82.Z cytomegaloviral disease, unspecified | 1D82.Y | 1D82.Y (5) | The anchor term "Cytomegaloviral pneumonitis" specifies a particular subtype (pneumonitis) not explicitly listed in the options. According to Rule 3a, "other specified cytomegaloviral disease" (1D82Y) is the correct choice when the term indicates a specific manifestation not included in the provided subtypes. |
| Gingival and edentulous alveolar ridge lesions associated with trauma | 525.20 Unspecified atrophy of edentulous alveolar ridge  525.21 Minimal atrophy of the mandible  525.22 Moderate atrophy of the mandible  525.23 Severe atrophy of the mandible  525.24 Minimal atrophy of the maxilla  525.25 Moderate atrophy of the maxilla  525.26 Severe atrophy of the maxilla | None | 525.20 (1)  None (4) | **Reason for selection:**  The anchor term specifies "edentulous alveolar ridge lesions," which directly aligns with code 52520 ("Unspecified atrophy of edentulous alveolar ridge"). While the term also mentions trauma, the options do not include trauma-specific codes. Since 52520 is the only code explicitly referencing the alveolar ridge (a key component of the anchor term), it is the best match under Rule 2. Other codes focus on mandible/maxilla atrophy, which are not explicitly mentioned in the anchor term.  **Reason for Rejection:**  The anchor term specifies "lesions associated with trauma," which involves injury-related abnormalities. All options describe atrophy (a reduction in tissue size), not trauma-induced lesions. None of the options match the specific condition of the anchor term, triggering Rule 1. |
| Horizontal alveolar bone loss | 525.20 Unspecified atrophy of edentulous alveolar ridge  525.21 Minimal atrophy of the mandible  525.22 Moderate atrophy of the mandible  525.23 Severe atrophy of the mandible  525.24 Minimal atrophy of the maxilla  525.25 Moderate atrophy of the maxilla  525.26 Severe atrophy of the maxilla | None | 525.20 (3)  None (2) | **Reason for selection:**  The anchor term "horizontal alveolar bone loss" specifies a particular type of atrophy (horizontal), but none of the options explicitly mention "horizontal." The closest match is 52520 ("Unspecified atrophy of edentulous alveolar ridge"), which covers atrophy of the alveolar ridge in general. Since the options lack a code for "horizontal," Rule 3a applies: "unspecified" is chosen as the best available match.  **Reason for rejection:**  The anchor term "Horizontal alveolar bone loss" specifies a directional type of bone loss not captured by any of the provided options. The available options describe atrophy severity (minimal, moderate, severe) and location (mandible/maxilla), but none mention "horizontal" as a directional characteristic. Since the options lack specificity for the directional aspect of the anchor term, no code directly aligns with the specific condition, triggering Rule 1. |
| Other cholelithiasis with obstruction | 574.10 Calculus of gallbladder with other cholecystitis, without mention of obstruction  574.11 Calculus of gallbladder with other cholecystitis, with obstruction | None | 584.11 (5) | The anchor term specifies "obstruction," which is explicitly mentioned in option 57411. While the term "other cholecystitis" in the option differs from "other cholelithiasis" in the anchor term, the presence of obstruction aligns with the anchor term's key condition. Rule 2 prioritizes direct alignment with the specified condition (obstruction), making 57411 the best match. |
| Gastrostomy hemorrhage | 578.0 Hematemesis  578.1 Blood in stool  578.9 Hemorrhage of gastrointestinal tract, unspecified | None | 578.9 (5) | The anchor term "Gastrostomy hemorrhage" refers to bleeding originating from a gastrostomy site, which is a specific location within the gastrointestinal tract. None of the options explicitly mention gastrostomy hemorrhage. Option 5789 is the most general match for a gastrointestinal hemorrhage, as it is the only code that broadly encompasses hemorrhage from the GI tract. Since no more specific code exists in the list, 5789 is the best available match (Rule 2). |

1. **Examples of False Positive Cases Due to Explicit Source-to-Parent Mappings in the Ground Truth for ICD-9-CM to ICD-10-CM within the Disease of the Digestive System Chapter.**

Table 9. Example of false positive cases due to source-to-parent mappings in the ground truth for ICD-9-CM to ICD-10-CM within the Diseases of the Digestive System chapter.

| Source | Options | Ground Truth | Predicted |
| --- | --- | --- | --- |
| **530.10** Esophagitis, unspecified | K20.90: Esophagitis unspecified without bleeding  K20.91: Esophagitis unspecified with bleeding | K20.9 | K20.90 |
| 530.11, Reflux esophagitis | K21.00: Gastro-esophageal reflux disease with esophagitis without bleeding  K21.01: Gastro-esophageal reflux disease with esophagitis with bleeding | K21.0 | K21.00 |
| 530.12, Acute esophagitis | K20.90: Esophagitis unspecified without bleeding  K20.91: Esophagitis unspecified with bleeding | K20.9 | None |
| 530.19, Other esophagitis | K20.80: Other esophagitis without bleeding  K20.81: Other esophagitis with bleeding | K20.8 | K20.80 |
| 530.82, Esophageal hemorrhage | K22.81: Esophageal polyp  K22.82: Esophagogastric junction polyp  K22.89: Other specified disease of esophagus | K22.8 | None |
| 530.83, Esophageal leukoplakia | K22.81: Esophageal polyp  K22.82: Esophagogastric junction polyp  K22.89: Other specified disease of esophagus | K22.8 | None |
| 530.89, Other specified disorders of esophagus | K22.81: Esophageal polyp  K22.82: Esophagogastric junction polyp  K22.89: Other specified disease of esophagus | K22.8 | K22.89 |
| 540.0, Acute appendicitis with generalized peritonitis | K35.20: Acute appendicitis with generalized peritonitis without abscess**  K35.21: Acute appendicitis with generalized peritonitis with abscess | K35.2 | K35.20 |
| 540.1, Acute appendicitis with peritoneal abscess | K35.30: Acute appendicitis with localized peritonitis without perforation or gangrene  K35.31: Acute appendicitis with localized peritonitis and gangrene without perforation  K35.32: Acute appendicitis with perforation and localized peritonitis without abscess  K35.33: Acute appendicitis with perforation and localized peritonitis with abscess | K35.3 | K35.33 |
| 564.89, Other functional disorders of intestine | K59.81: Ogilvie syndrome  K59.89: Other specified functional intestinal disorders | K59.8 | K59.89 |
| 576.1, Cholangitis | K83.01: Primary sclerosing cholangitis  K83.09: Other cholangitis | K83.0 | K83.09 |

1. **Examples of the False Positive Cases Due to Incorrect Selection Instead of Rejecting All Options for ICD-10-CM to ICD-9-CM within the Disease of the Digestive System Chapter.**

Table 10. Examples of the false positive cases where the model incorrectly selected an option instead of rejecting all the options and return None, for ICD-10-CM to ICD-9-CM mapping within the Disease of the Digestive System chapter. * represents the option selected by the model.

| **Source** | **Options** |
| --- | --- |
| Gingival and edentulous alveolar ridge lesions associated with trauma | 525.20 Unspecified atrophy of edentulous alveolar ridge *  525.21 Minimal atrophy of the mandible  525.22 Moderate atrophy of the mandible  525.23 Severe atrophy of the mandible  525.24 Minimal atrophy of the maxilla  525.25 Moderate atrophy of the maxilla  525.26 Severe atrophy of the maxilla |
| Horizontal alveolar bone loss | 525.20 Unspecified atrophy of edentulous alveolar ridge *  525.21 Minimal atrophy of the mandible  525.22 Moderate atrophy of the mandible  525.23 Severe atrophy of the mandible  525.24 Minimal atrophy of the maxilla  525.25 Moderate atrophy of the maxilla  525.26 Severe atrophy of the maxilla |
| Diverticulum of appendix | 562.0 Diverticula of small intestine  562.00 Diverticulosis of small intestine (without mention of hemorrhage)  562.01 Diverticulitis of small intestine (without mention of hemorrhage)  562.02 Diverticulosis of small intestine with hemorrhage  562.03 Diverticulitis of small intestine with hemorrhage  562.1 Diverticula of colon *  562.10 Diverticulosis of colon (without mention of hemorrhage)  562.11 Diverticulitis of colon (without mention of hemorrhage)  562.12 'Diverticulosis of colon with hemorrhage  562.13 Diverticulitis of colon with hemorrhage |
| Toxic liver disease with chronic lobular hepatitis | 571.40 Chronic hepatitis, unspecified  571.41 Chronic persistent hepatitis  571.42 Autoimmune hepatitis  571.49 Other chronic hepatitis * |
| Calculus of bile duct with chronic cholangitis without obstruction | 574.40 Calculus of bile duct with other cholecystitis, without mention of obstruction *  574.41 Calculus of bile duct with other cholecystitis, with obstruction |
| Other cholelithiasis without obstruction | 574.10 Calculus of gallbladder with other cholecystitis, without mention of obstruction *  574.11 Calculus of gallbladder with other cholecystitis, with obstruction |
| Other cholelithiasis with obstruction | 574.10 Calculus of gallbladder with other cholecystitis, without mention of obstruction  574.11 Calculus of gallbladder with other cholecystitis, with obstruction * |
| Gastrostomy hemorrhage | 578.0 Hematemesis  578.1 Blood in stool  578.9 Hemorrhage of gastrointestinal tract, unspecified * |

1. **Detailed Evaluation of the Prompting Framework.**

We analysed the performance of **PR** using four key outcomes: correct selections (*True Positives*), correct rejections (*True Negatives*), incorrect selections (*False Positives*), and incorrect rejections (*False Negatives*). As shown in *Table 10*, the **PR** improved correct mappings and filtered out invalid ones, though some false positives and false negatives remained.

Table 11. Evaluation of the prompting framework (**PR**) for handling hierarchical inconsistencies across ICD versions. Results show the average number (± standard deviation) of correct selections (True Positive), correct rejections (True Negative), incorrect selections (False Positive), and incorrect rejections (False Negative) cases for the HA+LG method with Qwen3-8B, averaged over five runs.

|  | **ICD-9-CM to ICD-10-CM** | | | **ICD-10-CM to ICD-9-CM** | | | **ICD-10-AM to ICD-11** | | | **ICD-11 to ICD-10-AM** | | |
| --- | --- | --- | --- | --- | --- | --- | --- | --- | --- | --- | --- | --- |
|  | **Dig** | **Inf** | **Resp** | **Dig** | **Inf** | **Resp** | **Dig** | **Inf** | **Resp** | **Dig** | **Inf** | **Resp** |
| TP | 42.4  ±2.58 | 54.2  ±2.14 | 11.2  ±0.98 | 62.6  ±1.96 | 0.0  ±0.0 | 18.2  ±1.6 | 21.2  ±1.33 | 21.8  ±1.94 | 11.6  ±1.2 | 23.6  ±2.94 | 41.8  ±1.17 | 4.8  ±0.98 |
| TN | 8.6  ±0.8 | 4.4  ±1.02 | 0.4  ±0.49 | 5.6  ±1.36 | 0.0  ±0.0 | 3.4  ±1.36 | 4.4  ±1.02 | 3.8  ±0.4 | 0.4  ±0.49 | 10.0  ±1.67 | 4.4  ±0.8 | 2.0  ±0.89 |
| FP | 13.6  ±1.02 | 13.8  ±1.94 | 8.4  ±1.2 | 13.6  ±1.94 | 0.0  ±0.0 | 7.4  ±2.58 | 19.6  ±3.5 | 36.0  ±2.1 | 12.2  ±1.94 | 26.6  ±3.5 | 20.4  ±1.74 | 6.6  ±1.5 |
| FN | 2.0  ±0.89 | 2.4  ±0.8 | 0.0  ±0.0 | 2.4  ±0.8 | 0.0  ±0.0 | 4.8  ±0.4 | 15.2  ±1.72 | 9.6  ±2.33 | 0.2  ±0.4 | 15.2  ±1.72 | 4.0  ±1.26 | 0.8  ±0.75 |
| **Total** | 66.6  ±2.33 | 74.8  ±3.71 | 20.0  ±1.41 | 84.0  ±2.19 | 0.0  ±0.0 | 33.8  ±3.06 | 49.4  ±1.36 | 71.2  ±0.98 | 24.4  ±2.06 | 75.4  ±4.54 | 70.6  ±2.73 | 14.2  ±1.33 |
